# The Puzzling Unidimensionality of the DSM Substance Use Disorders: Commentary

**DOI:** 10.3389/fpsyt.2013.00156

**Published:** 2013-11-26

**Authors:** Christopher Stephen Martin

**Affiliations:** ^1^Western Psychiatric Institute and Clinic, University of Pittsburgh School of Medicine, Pittsburgh, PA, USA

**Keywords:** diagnosis, DSM-5, substance use disorders, unidimensionality, addiction

This article raises a number of interesting issues regarding the diagnosis and the very nature of substance use disorders (SUDs) (1). The field has much to learn about whether SUDs and their symptoms are best understood in terms of reflective, formative, network, or other models. These issues are important in other areas of psychiatric diagnosis as well. However, studying such models of psychopathology might increase our knowledge of the etiology and clinical course of disorders, far more than fundamentally changing and improving the nature of our diagnostic system.

DSM-5 does not articulate any specific model of how SUDs are related to SUD symptoms. In DSM-5, for each substance class, there is a single category of symptoms that defines a single SUD. This explicitly casts SUD symptoms as part of a single category or dimension, and in this sense the criteria are “unidimensional.” However, this term can mean different things: the unidimensionality of DSM-5 SUD criteria does not imply a reflective model of psychopathology, nor does it convey specific information about the nature or coherence of any underlying latent construct or constructs. While the diagnostic entity of SUD is a single super-ordinate category, SUD is not a single coherent latent construct. Instead SUD symptoms reflect a variety of rather distinct addiction constructs and phenomena, such as craving, withdrawal, negative consequences, and compulsive patterns of substance use. The symptoms were intended to provide non-overlapping information, rather than being interchangeable items sampled from a broad domain, such as on a vocabulary test. While SUD symptoms and the various constructs they were designed to measure are conceptually distinct, they all tend to be moderately inter-correlated with each other. That is, they form a single, yet broad and loose, super-ordinate dimension. This situation is common in psychiatric diagnosis.

The article notes that most factor analyses that have found evidence for a single broad dimension of SUD symptoms have been mathematically specified using a reflective model. But this does not mean that DSM endorses or is based on a reflective model. Indeed, as the article points out, latent factor and similar analyses can (and should) be alternatively specified using the assumptions of formative and other models. The more general point is that none of the six models in Figure 1 are inconsistent with the idea that SUDs can be diagnosed with a single criterion array if the criteria are associated with each other. Note that there is only one latent construct in Figure 1B, only one network in Figure 1F, and so forth. Whether emerging knowledge favors reflective, formative, network, or other models for SUDs, it is likely the case that in the future SUD diagnosis will still involve meeting X or more out of a set of Y criteria.

Beyond diagnosis, studying reflective, formative, network, and other models of SUDs is important and promises to tell us more about the very nature of addiction. Little is known about whether and under what conditions SUD symptoms and addiction constructs can causally influences other SUD symptoms and addiction constructs, and little research has addressed network models of SUDs. Better understanding of how substance problems can influence other substance problems will increase knowledge of etiology and clinical course, and may suggest novel treatment targets.

## References

[1] MacCoun RJ The puzzling unidimensionality of DSM-5 substance use disorder diagnoses. Front Psychiatry (2013) 4:153 10.3389/fpsyt.2013.00153 PMC3839091 24324446

